# Both Phenolic and Non-phenolic Green Tea Fractions Inhibit Migration of Cancer Cells

**DOI:** 10.3389/fphar.2016.00398

**Published:** 2016-11-03

**Authors:** Ean-Jeong Seo, Ching-Fen Wu, Zulfiqar Ali, Yan-Hong Wang, Shabana I. Khan, Larry A. Walker, Ikhlas A. Khan, Thomas Efferth

**Affiliations:** ^1^Department of Pharmaceutical Biology, Institute of Pharmacy and Biochemistry, Johannes Gutenberg UniversityMainz, Germany; ^2^National Center for Natural Products Research, School of Pharmacy, University of MississippiOxford, MS, USA; ^3^Department of BioMolecular Sciences, School of Pharmacy, University of MississippiOxford, MS, USA

**Keywords:** chemoprevention, green tea, microarray, nutrigenomics, theaceae

## Abstract

Green tea consumption is associated with chemoprevention of many cancer types. Fresh tea leaves are rich in polyphenolic catechins, which can constitute up to 30% of the dry leaf weight. While the polyphenols of green tea have been well investigated, it is still largely unknown, whether or not non-phenolic constituents also reveal chemopreventive and anti-metastatic effects. In this study, we investigated the effects of a fraction of green tea rich in phenolic compounds (PF), a non-phenolic fraction (NPF), which contains glyceroglycolipids (GGL), and a pure glyceroglycolipid compound isolated from the non-phenolic fraction in human cancer. Dried green tea leaves were extracted and applied to a Sephadex LH-20 column. The resazurin reduction assay was used to investigate the cytotoxicity of green tea samples toward human HepG2 hepatocellular carcinoma and normal AML12 hepatocytes cells. Gene expression profiling was performed by mRNA microarray hybridization and the microarray results were validated by RT-PCR. The scratch migration assay was used to investigate the effects of green tea samples on cell migration *in vitro*. The changes of microtubule dynamics were observed using fluorescence microscopy. PF and NPF were prepared from methanol extract of green tea. A GGL was isolated from NPF. All three green tea samples did not show significant cytotoxic activity up to 10 μg/mL in both HepG2 and AML12 cells, whereas cytotoxicity of the control drug doxorubicin was observed with both cell lines (IC_50_ on AML12: 0.024 μg/mL, IC_50_ on HepG2: 2.103 μg/mL). We identified three sets of genes differentially expressed upon treatment with the green tea samples. The genes were associated with cytoskeleton formation, cellular movement, and morphology. The correlation coefficients between mRNA expression values determined by microarray and RT-PCR were *R* = 0.94. HepG2 and U2OS cells treated with green tea extracts showed the delayed closures. Besides, the number of distinct tubulin filaments decreased upon treatment with green tea samples. We identified not only PF, but also glyceroglycolipids in NPF as contributing factors to the chemopreventive effects of green tea. Both PF and NPF of green tea inhibited cancer cell migration by the disassembly of microtubules, even though they were not cytotoxic.

## Introduction

Tea is one of the most popular beverages around the world. It is obtained from the leaves of *Camellia sinensis* L. Kuntze (Theaceae) as green, black, or oolong tea (Balentine et al., 1997; Jankun et al., 1997). Fresh tea leaves are rich in polyphenols known as catechins, which may constitute up to 30% of the dry leaf weight (Cabrera et al., 2006; Chacko et al., 2010). The most prominent catechins are epicatechin, epicatechin-3-gallate, epigallocatechin, and epigallocatechin-3-gallate (EGCG) (Sano et al., 2001; Cabrera et al., 2006; Chacko et al., 2010). Other polyphenols include flavonols and their glycosides and one compound unique to tea, theogallin (3-galloylquinic acid) (Saleh et al., 2013). Green tea leaves contain three main classes of compounds, which are known to affect human health, i.e., xanthic bases (caffeine and theophylline), essential oils, and polyphenolic compounds (Graham, 1992). Caffeine is present at an average level of 3% along with very small amounts of the other common methylxanthines, theobromine, and theophylline (Graham, 1992). The amino acid theanine (N^5^-ethylglutamine) is also unique to tea (Graham, 1992). However, the nonphenolic components of green tea have not been explored in detail and their biological effects are unknown.

Several biological properties have been reported for green tea such as anti-inflammatory, anti-arthritic, antimicrobial, anti-oxidative, neuroprotective, antidiabetic, anti-angiogenic, and anticancer effects (Chacko et al., 2010; Hosseini and Ghorbani, 2015). Green tea consumption has been linked to the prevention of many types of cancer, including those of lung, colon, esophagus, mouth, stomach, small intestine, kidney, pancreas, and mammary glands (Chacko et al., 2010). Catechins are considered to be responsible for most of the biological properties of green tea (Bettuzzi et al., 2006; Chacko et al., 2010). In a double-blind, placebo-controlled study, green tea catechins were safe and effective for treating premalignant prostate cancer (Bettuzzi et al., 2006). Several other studies also support the protective effects of green tea against prostate, esophageal, colon, rectum, and pancreatic cancers (Ji et al., 1997; Jian et al., 2004). A protective effect of green tea against liver injury was also supported by animal studies (Arteel et al., 2002; Abe et al., 2005, 2007).

Tea polyphenols are in general considered as strong antioxidants, and the anti-oxidative activity of tea polyphenols has been linked with decreased oxidative DNA damage. For example, supplementation of the diet of heavy smokers with green tea reduced urinary 8-hydroxydeoxy-2′-deoxyguanosine (8-OHdG) compared with the control group (Hakim et al., 2003; Schwartz et al., 2005). EGCG serves as hydrogen bond donor and binds many proteins, e.g., fibronectin, fibrinogen and histidine-rich glycoproteins, laminin receptor and Bcl-2 (Leone et al., 2003; Tachibana et al., 2004; Umeda et al., 2008). EGCG inhibited the phosphorylation of JNK (JUN N-terminal kinase), JUN, MEK1, MEK2, ERK1, ERK2, and ELK1 (Ets-like protein 1) in JB6 epidermal cells (Dong et al., 1997; Chung et al., 1999, 2001). Besides, several studies demonstrated the inhibitory effects of EGCG on the EGFR signaling pathways (Liang et al., 1997; Hou et al., 2005; Shimizu et al., 2005; Adachi et al., 2007, 2008). Inhibition of EGFR signaling also decreased VEGF A expression in cancer cells (Masuda et al., 2002). These investigations clearly demonstrate that the cancer-preventive effects of EGCG are caused by multiple molecular mechanisms.

According to the multi-step model of carcinogenesis, tumors develop in three main steps: (1) initiation, where persistent DNA lesions occur, (2) proliferation stimuli in the promotion phase, and (3) the progression phase, which includes genomic instability, metastasis and neo-angiogenesis. Green tea and EGCG inhibit not only initiation and promotion of the carcinogenic process, but also progression (Park and Surh, 2004; Rathore and Wang, 2012). While the polyphenols of green tea have been well investigated, it is still largely unknown, whether or not non-phenolic constituents also reveal chemopreventive and anti-metastatic effects. Inhibition of invasion and cell migration by EGCG as initial steps of metastasis have been reported for cell lines of diverse types (Khan and Mukhtar, 2010). Less is known, however, about inhibition of migration of liver cancer cells by green tea extracts and the underlying mechanisms are not understood in this tumor entity. Since hepatocellular carcinoma has a high incidence in Southeast Asia (Goh et al., 2015) and green tea is very popular in entire Asia, the exploration of the chemopreventive effects of green tea extracts and their molecular modes of action against liver cancer represents an obvious and important topic.

In this study, we investigated possible targets and mechanisms of chemopreventive action of green tea in human HepG2 hepatocellular carcinoma cells. Our study focused on investigating the efficacy of a fraction of green tea rich in phenolic compounds, a non-phenolic fraction, which contains glyceroglycolipids, and a pure compound belonging to glyceroglycolipid class, which was isolated from the non-phenolic fraction. For the systematic and comprising search for underlying modes of action of green tea constituents, we compared microarray-based, transcriptome-wide mRNA expression profiles of treated vs. untreated cells. Differentially expressed genes were subjected to pathway profiling and the identified pathways were subsequently experimentally verified by independent experimental methods.

## Materials and methods

### Preparation of green tea samples

Dried green tea leaves of Chirag Trade Mark were purchased in Memphis TN. A reference (No. 4915) have been deposited at the National Center for Natural Products Research, University of Mississippi. The leaves were extracted with methanol at room temperature. The dried methanol extract (28.8% w/w) was partitioned between hexanes and methanol. After removal of chlorophylls, the methanol extract was applied to a Sephadex LH-20 column and eluted with methanol. The resultant fractions were separated into non-phenolic fraction (NPF, 4.5% w/w) and phenolic fraction (PF, 18.7% w/w) based on TLC profiles. GGL, a glyceroglycolipid was isolated from NPF.

### NMR and mass data analysis

NMR spectra were recorded in C_5_D_5_N on a Varian AS 400 or Varian Unity Inova 600 NMR spectrometers. HRESIMS data were obtained on an Agilent Series 1100 SL mass spectrometer.

### Analysis conditions for catechin standards and phenolic fraction of green tea methanol extract

The analysis was performed on a Waters Acquity UPLC system (Waters Corp., Milford, MA) that included a binary solvent manager, sampler manager, heated column compartment, photo-diode array (PDA) detector, and single quadrupole detector (SQD). The instrument was controlled by Waters Empower 2 software. A Waters UPLC Shield RP18 column (2.1 × 100 mm I.D., 1.7 μm) was used. The column and sample temperatures were maintained at 35°C and 10°C, respectively. The eluent consisted of water containing 0.05% formic acid (A) and acetonitrile with 0.05% formic acid (B). The analysis was performed using the following gradient elution at a flow rate of 0.25 mL/min: 0–2.0 min, held at 2% B; 2.0–3.0 min, 2% B to 7% B; and 3.0–15.0 min, 7% B to 25% B. The analysis was followed by a 3 min washing procedure with 100% B and re-equilibration period of 4.5 min. All solutions were filtered through 0.45 μm PTFE filters. The injection volume was 2 μL. The PDA detection wavelength was 230 nm. An ESI source was used in the positive mode. The source temperature and the desolvation gas temperature were maintained at 150°C and 350°C, respectively. The probe voltage (capillary voltage), cone voltage, and extractor voltage were fixed at 3.5 kV, 30 V, and 3.0 V, respectively. Nitrogen was used as the desolvation gas (650 L/h) and drying gas (25 L/h). Mass spectra were obtained at 500 Da/s scan rate.

### Analysis conditions for glyceroglycolipd standards and non-phenolic fraction of green tea methanol extract

The analysis was performed on a Waters Acquity UPLC system (Waters Corp., Milford, MA) that included a binary solvent manager, sampler manager, heated column compartment, PDA detector, and single quadrupole detector (SQD). The instrument was controlled by Waters Empower 2 software. A Waters Cortec UPLC C18 column (2.1 × 100 mm I.D., 1.6 μm) was used. The column and sample temperatures were maintained at 35°C and 10°C, respectively. The eluent consisted of water containing 0.05% formic acid (A) and acetonitrile with 0.05% formic acid (B). The analysis was performed using the following gradient elution at a flow rate of 0.25 mL/min: 0–7.0 min, 55% B to 95% B; 7.0–8.0 min, 95% B to 100% B; and held at 100% B in next 4 min. The analysis was followed by a re-equilibration period of 4.5 min. All solutions were filtered through 0.45 μm PTFE filters and the injection volume was 5 μL. The PDA detection wavelength was 200 nm. An ESI source was used in the positive mode. The source temperature and the desolvation gas temperature were maintained at 150 and 350°C, respectively. The probe voltage (capillary voltage), cone voltage, and extractor voltage were fixed at 3.0 kV, 30 V, and 3.0 V, respectively. Nitrogen was used as the desolvation gas (650 L/h) and drying gas (25 L/h). Mass spectra were obtained at 500 Da/s scan rate.

### Resazurin reduction assay

The resazurin reduction assay was used to investigate the cytotoxicity of green tea samples toward human HepG2 hepatocellular carcinoma and normal AML12 hepatocytes cells. The assay is based on reduction of the indicator dye, resazurin, to the highly fluorescent resorufin by viable cells. Non-viable cells rapidly lose the metabolic capacity to reduce resazurin and, thus, do not produce fluorescent signal (O'Brien et al., 2000; Seo et al., 2015). Briefly, adherent cells were detached by 0.25% trypsin/EDTA (Invitrogen, Darmstadt, Germany) and 5000 cells were placed in each well of a 96-well cell culture plate (Thermo Scientific, Schwerte, Germany) in a total volume of 100 μL. Cells were attached overnight and then were treated with different concentrations of test samples. After 72 h incubation, 20 μL resazurin (Sigma-Aldrich, Taufkirchen, Germany) 0.01% w/v in ddH_2_O was added to each well and the plates were incubated at 37°C for 4 h. Fluorescence was measured by an Infinite M2000 Proplate reader (Tecan, Crailsheim, Germany) using an excitation wavelength of 544 nm and an emission wavelength of 590 nm. Each experiment was done at least three times, with six replicates each. The cell viability was calculated as percentage of untreated control.

### mRNA microarray

HepG2 cells were seeded and incubated for 24 h prior to treatment with green tea sample (PF, NPF, or GGL). Cells were treated with 25 μg/mL of the test sample or DMSO as solvent control (0.5%) for 24 h. Then, total RNA was isolated using InviTrap Spin Universal RNA Mini kit (250) (Stratec Molecular, Berlin, Germany). The experiment was performed in duplicates for treated samples and for control samples at the Institute for Molecular Biology (IMB) as previously described (Wiench et al., 2012). The quality of total RNA was confirmed by gel analysis using the total RNA Nano chip assay on an Agilent 2100 Bioanalyzer (Agilent Technologies, Berlin, Germany). Only samples with RNA index values greater than 9.3 were selected for expression profiling. RNA concentrations were determined using the NanoDrop spectrophotometer (NanoDrop Technologies, Wilmington, DE). Biotin-labeled cRNA samples for hybridization on Illumina Human Sentrix-HT12 BeadChip arrays (Illumina, Inc., San Diego, CA, USA) were prepared according to Illumina's recommended sample labeling procedure based on the modified Eberwine protocol (Eberwine et al., 1992). In brief, 250–500 ng total RNA was used for complementary DNA (cDNA) synthesis, followed by an amplification/labeling step (*in vitro* transcription) to synthesize biotin-labeled cRNA according to the Message Amp II a RNA Amplification kit (Ambion, Inc., Austin, TX). Biotin-16-UTP was purchased from Roche Applied Science (Penzberg, Germany). The cRNA was column purified according to Total Prep RNA Amplification Kit, and eluted in 60–80 μL of water. The quality of cRNA was controlled using the RNA Nano Chip Assay on an Agilent 2100 Bioanalyzer and spectrophotometrically quantified (NanoDrop). Subsequent hybridization was performed according to the manufacturer's instructions. Microarray scanning was done using a Beadstation array scanner, setting the adjustment to a scaling factor of 1 and photomultiplier tube settings at 430. Data extraction was performed for all beads individually, and outliers were removed, if the median absolute deviation exceeded 2.5. Then, mean average signals and standard deviations were calculated for each probe. Data analysis was done by normalization of signals using the quantile normalization algorithm without background subtraction. Differentially regulated genes were defined by calculating the standard deviation differences of a given probe in a one-by-one comparison of samples or groups. The data was further processed using Chipster software (The Finnish IT Center for Science CSC, Espoo, Finland).

### Real-time reverse transcription PCR

Real-time RT-PCR was performed with the same samples that were used for microarray experiments. Total RNA was isolated as described before and converted to cDNA with random hexamer primers using RevertAid H Minus First Strand cDNA Synthesis kit (Thermo Scientific, Waltham, MA, USA). PCR primers for five genes (Table 1) were designed using Roche Universal Probe Design (http://www.rocheapplied-science.com/sis/rtpcr/upl/index.jsp?id=UP030000) and GenScript Real Time PCR Primer Design (https://www.genscript.com/ssl-bin/app/primer) tools. Amplification specificities were checked with Primer Blast (http://www.ncbi.nlm.nih.gov/tools/primer-blast) using the sequence data from the NCBI RefSeq Human mRNA database. Oligonucleotides were synthesized by Eurofins MWG Operon (Ebersberg, Germany). Primer sequences are shown in Table 1. Real-time RT-PCR experiments were performed on CFX384 Real-Time PCR Detection System (Bio-Rad, Munich, Germany). Four microliters of 5 × Hot Start Taq EvaGreen qPCR Mix (no ROX) (Axon, Kaiserslautern, Germany), 250 nM final primer concentration and 300 ng RNA (converted to cDNA) were used per reaction. RT-PCR was performed as follows: 50°C for 2 min, initial denaturation at 95°C for 10 min, 40 cycles including strand separation at 95°C for 15 s, annealing at 56.5°C for 1 min, and extension at 72°C for 1 min following final extension at 72°C for 1 min. The housekeeping gene *RPS13* served as reference for standardization. All measurements were performed in duplicates. Standardized C_t_ (cycle threshold) values for the genes in samples were obtained by dividing the C_t_ values of genes in drug-treated samples by C_t_ values of *RPS13* gene and multiplying with the C_t_ value of *RPS13* in the DMSO control. Fold changes were calculated with the ΔC_t_ (standardized C_t_ of the gene in drug-treated sample—C_t_ of the gene in DMSO control) method where the fold change is equal to 2^−ΔCt^ for the up-regulated genes and −(2^ΔCt^) for the down-regulated genes.

**Table 1:** **Primer nucleotide sequences used for real-time RT-PCR experiments**.

**Target gene**	**Primer sequences**
*CD86*	Fw: 5′- ATTCTGAACTGTCAGTGCTTGC -3′
	Rev: 5′- CTTCTTAGGTTCTGGGTAACCG -3′
*ZNF365*	Fw: 5′- GTTTGGCGTTGGCAGTCAGGTAAT -3′
	Rev: 5′- CACAGCACGACTCTGCAAGTGTAT -3′
*SYN1*	Fw: 5′- AAC AGG CCG AAT TCT CTG AT -3′
	Rev: 5′- CCA TTC CGA AGA ACT TCC AT -3′
*STK11*	Fw: 5′- TGA CTG TGG TGC CGT ACT TG -3′
	Rev: 5′- CAC CGT GAA GTC CTG AGT GT -3′
*RPS13*	Fw: 5′- GGTTGAAGTTGACATCTGACGA -3′
	Rev: 5′- CTTGTGCAACACATGTGAAT -3′

### Ingenuity pathway analysis

Microarray data were analyzed through the use of IPA (Ingenuity® Systems, www.ingenuity.com). IPA software relies on the Ingenuity Knowledge Base, a timely updated database containing biological and chemical interactions and functional annotations gathered from literature. In order to get information about cellular functions, networks and affected pathways, the Core Analysis tool of IPA was used for all datasets.

### Scratch migration assay

The scratch migration assay was used to investigate the effects of green tea samples on cell migration *in vitro* (Liang et al., 2007). Briefly, 2 × 10^6^ HepG2 cells or 1 × 10^6^ U2OS-GFP-α-tubulin cells were seeded in each well of a 6-well plate and allowed to grow to a confluent monolayer for 24 h. The cell monolayer was carefully scraped with a sterile p200 pipet tip to create a scratch. Subsequently, cells were washed with PBS and DMEM culture medium containing 25 μg/mL of green tea sample (PF, NPF, or GGL) or DMSO (solvent control) was added and incubated further. Images of the scratches were taken after 48 h using a Juli Br live cell analyzer (VWR International, Erlangen, Germany) at 10 × magnification. Data analysis was performed by TScratch software (Gebäck et al., 2009).

### Imaging of structure and dynamics of the microtubule cytoskeleton by fluorescence microscopy

2 × 10^4^ U2OS-GFP-α-tubulin cells were seeded in each well of a sterile ibi Treat μ-slide (ibidi, Germany) and cells were allowed to attach overnight. Cells were treated with 25 μg/mL of green tea sample (PF, NPF, or GGL) or DMSO (solvent control) and incubated at 37°C for 2 h. After rinsing with PBS and staining for 15 min with 300 nM of 4′,6-diamidino-2-phenylindole (DAPI) (Life Technologies, Darmstadt, Germany), the cells were washed with PBS and mounted. Fluorescence imaging was performed by using 470 nm excitation and 525 nm emission for GFP and 360 nm excitation and 447 nm emission for DAPI of EVOS digital inverted microscope (Life Technologies). Each experiment was repeated at least three times and representative images were selected.

## Results

### Green tea samples

**Figure 1:**
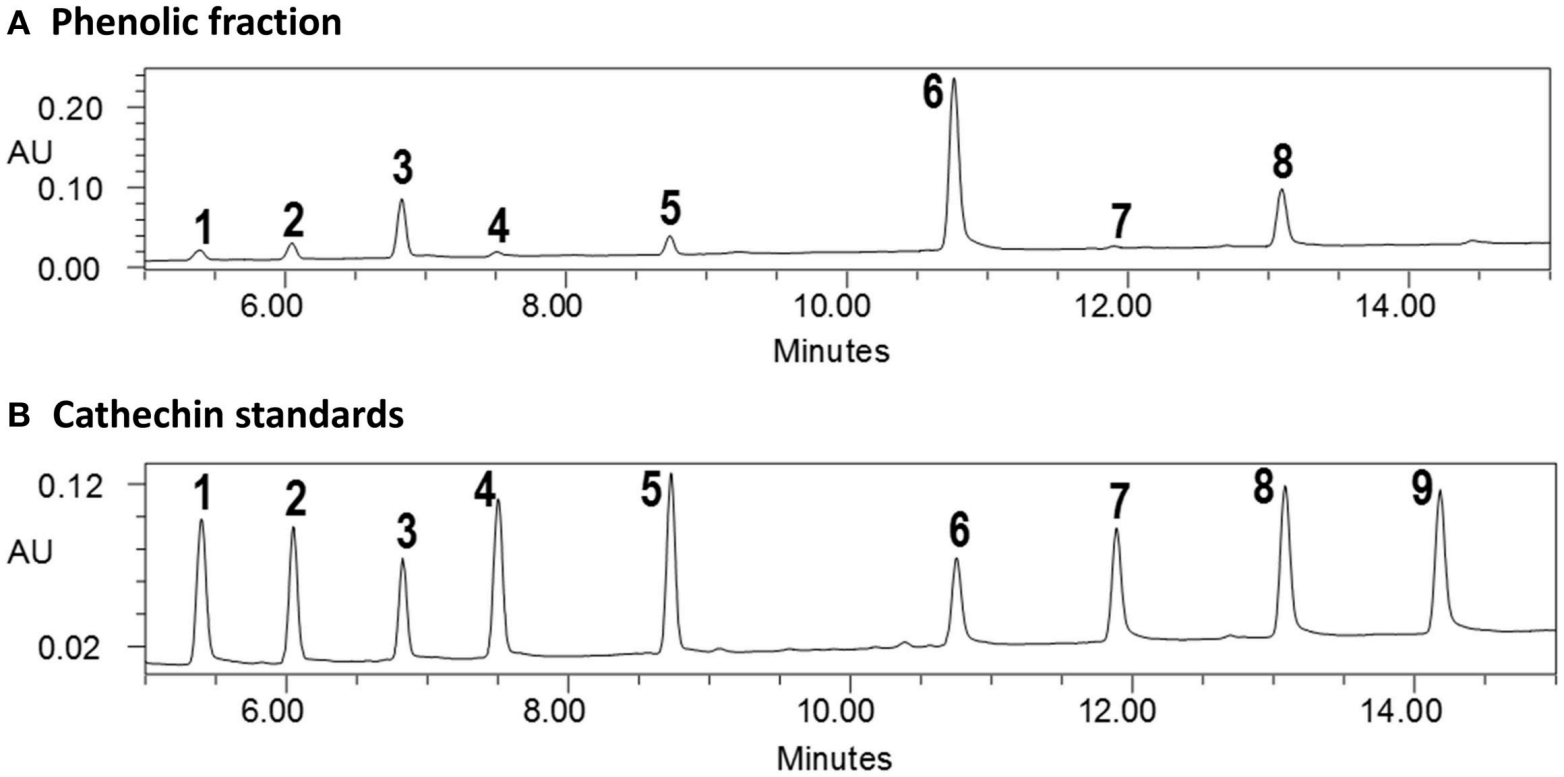
**UPLC-UV analysis of (A) phenolic fraction and (B) catechins standards (cross reference with Table 2)**.

**Figure 2:**
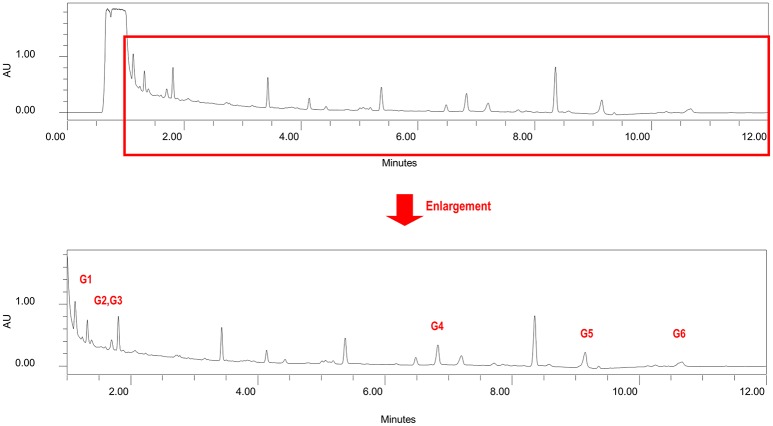
**Chromatogram of non-phenolic fraction (cross reference with Table 3)**.

Several glyceroglycolipids (2.0% of non-phenolic fraction, NPF) were isolated from NPF. The isolated compounds were identified, by NMR and mass data, as 3-[(1-oxohexadecyl)oxy]-2-[(1-oxooctadecyl)oxy]propyl-6-*O*-α-D-galactopyranosyl β-D-galactopyranoside (GGL, 0.142% of NPF); gingerglycolipid A (0.172% of NPF); (2*S*)-2-hydroxy-3-[(1-oxohexadecyl)oxy]propyl 6-*O*- α-D-galactopyranosyl-β-D-galactopyranoside (0.021% of NPF); (2*S*)-2-hydroxy-3-[[(9Z,12Z,15Z)-1-oxo-9,12,15-octadecatrienyl]oxy]propyl-β-D-galactopyranoside (0.11% of NPF); (2*S*)-2,3-bis[[(9Z,12Z,15Z)-1-oxo-9,12,15-octadecatrienyl]oxy]propyl 6-*O*-α-D-galactopyranosyl-β-D-galactopyranoside (0.436% of NPF); (2*S*)-2-[(1-oxohexadecyl)oxy]-3-[[(9*Z*,12*Z*,15*Z*)-1-oxo-9,12,15-octadecatrienyl]oxy]propyl 6-*O*-α-D-galactopyranosyl-β-D-galactopyranoside (0.645% of NPF); (2*S*)-2-[[(9*Z*,12*Z*,15*Z*)-1-oxo-9,12,15-octadecatrien-1-yl]oxy]-3-[(1-oxooctadecyl)oxy]propyl 6-*O*-α-D-galactopyranosyl-β-D-galactopyranoside (0.105% of NPF); and (2*S*)-2-[(1-oxohexadecyl)oxy]-3-[[(9Z,12Z,15Z)-1-oxo-9,12,15-octadecatrien-1-yl]oxy]propyl 6-deoxy-6-sulfo-α-D-glucopyranoside (0.130% of NPF). One of the isolated glyceroglycolipids (GGL) was selected to be included in this study. Eight catechins and caffeine were identified in PF by UHPLC-UV-MS [(−)-gallocatechin (1.83% of Phenolic Franction, (PF)), caffeine (2.71% of PF), epigallocatechin (14.5% of PF), catechin (0.40% of PF), (−)-epicatechin (2.23% of PF), epigallocatechin gallate (46.9% of PF), gallocatehin gallate (0.81% of PF), epicatechin gallate (8.55% of PF), and (−)-catechin gallate (0.10% of PF)] (Figures 1, 2, Tables 2, 3).

**Table 2:** **Contents (% = mg/100 mg sample) of catechins and caffeine in phenolic fraction**.

**No**.	**Compound name**	**% in PF**
1	(–)-Gallocatechin	1.83
2	Caffeine	2.71
3	Epigallocatechin	14.5
4	Catechin	0.40
5	(–)-Epicatechin	2.23
6	Epigallocatechin gallate	46.9
7	Gallocatehin gallate	0.81
8	Epicatechin gallate	8.55
9	(–)-Catechin gallate	0.10

**Table 3:** **Contents of six glyceroglycolipid standards in non-phenolic fraction NPF**.

**No**.	**Compound name**	**% in NPF**
G1	Gingerglycolipid A	0.172
G2	(2*S*)-2-Hydroxy-3-[(1-oxohexadecyl)oxy]propyl 6-*O*-α-D-galactopyranosyl-β-D-galactopyranoside	0.021
G3	(2*S*)- 2-Hydroxy-3-[[(9*Z*,12*Z*,15*Z*)- 1-oxo-9,12,15-octadecatrienyl]oxy]propyl-β-D-galactopyranoside	0.11
G4	(2*S*)-2,3-Bis[[(9*Z*,12*Z*,15*Z*)-1-oxo-9,12,15-octadecatrienyl]oxy]propyl 6-*O*-α-D-galactopyranosyl-β-D-galactopyranoside	0.436
G5	(2*S*)-2-[(1-Oxohexadecyl)oxy]-3-[[(9*Z*,12*Z*,15*Z*)-1-oxo-9,12,15-octadecatrienyl]oxy]propyl 6-*O*-α-D-galactopyranosyl-β-D-galactopyranoside	0.645
G6	(2*S*)-2-[[(9*Z*,12*Z*,15*Z*)-1-Oxo-9,12,15-octadecatrien-1-yl]oxy]-3-[(1-oxooctadecyl)oxy]propyl 6-*O*-α-D-galactopyranosyl-β-D-galactopyranoside	0.105

### Cytotoxic effect of PF, NPF, and GGL and their combinations (PF+NPF, PF+GGL, NPF+GGL, and PF+NPF+GGL) toward HepG2 cancer cells and AML12 normal hepatocytes

**Figure 3:**
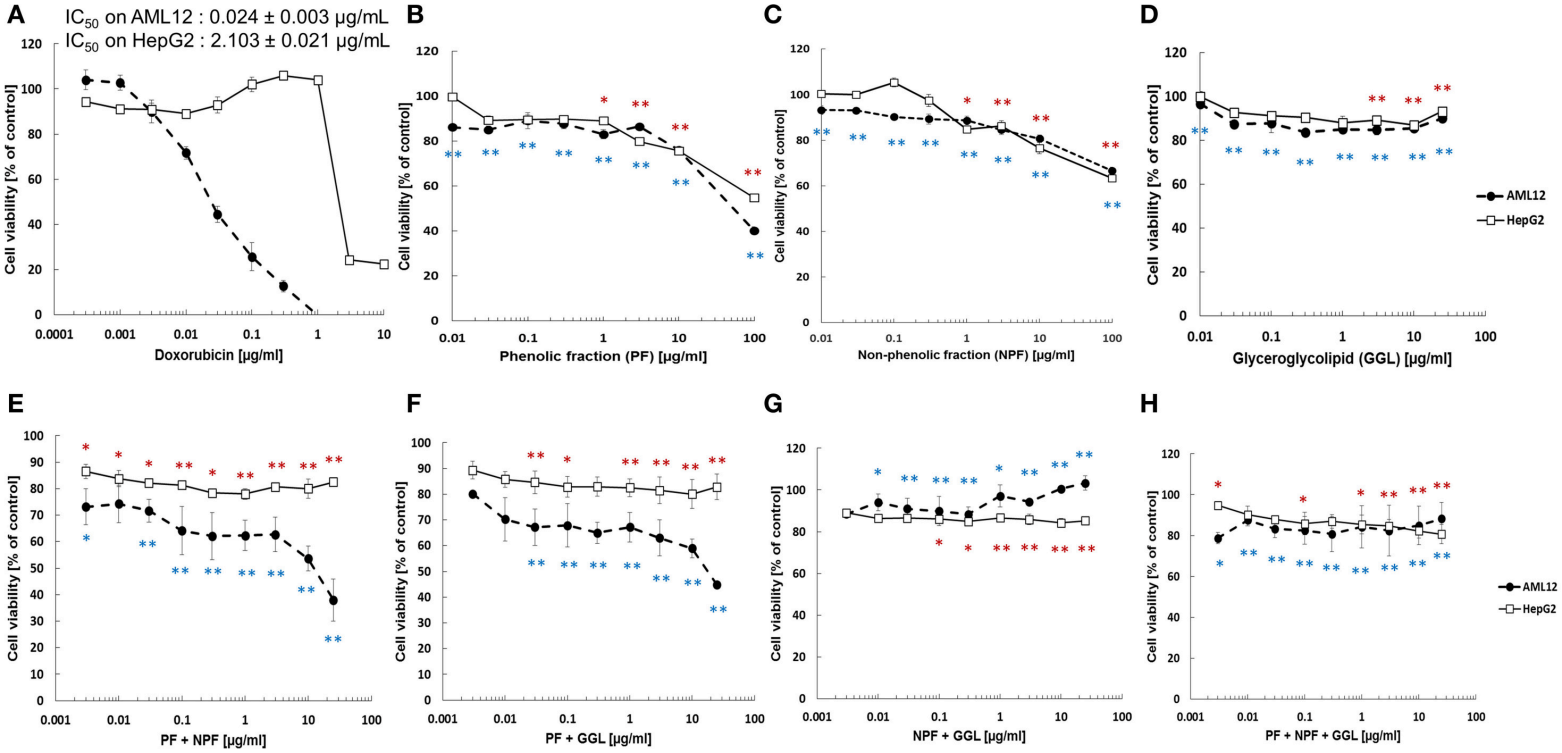
**Cytotoxicity of green tea samples (A) doxorubicin, (B) PF, (C) NPF, (D) GGL, (E) PF+NPF, (F) PF+GGL, (G) NPF+GGL, and (H) PF+NPF+GGL toward HepG2 hepatocellular carcinoma cells and AML12 normal hepatocytes**. Significantly different between cell viability of doxorubicin and cell viability of tested samples according to Student's *t*-test, ^*^0.01 < *P* ≤ 0.05, ^**^P ≤ 0.01, blue for AML12 and red for HepG2 cells.

In order to study the cytotoxicity of green tea samples toward human hepatocellular carcinoma, HepG2 cells were treated with PF, NPF, GGL or their combinations (PF+NPF, PF+GGL, NPF+GGL, and PF+NPF+GGL) for 72 h up to a highest concentration of 100 μg/mL (PF and NPF), 25 μg/mL (GGL), 12.5 μg/mL of each sample for two sample combinations (PF+NPF, PF+GGL, NPF+GGL) and 8.3 μg/mL of each sample in three extract combinations. Doxorubicin was used as cytotoxic control drug in our resazurin reduction assay in the range of 0.003–10 μg/mL in order to compare its cytotoxicity with those of green tea extracts. The effects in HepG2 cancer cells were compared with those in AML12 normal hepatocytes. All three green tea samples did not show significant cytotoxic activity up to 10 μg/mL in the resazurin assay, whereas cytotoxicity of doxorubicin was observed with both cell lines (IC_50_ on AML12: 0.024 ± 0.003 μg/mL, IC_50_ on HepG2: 2.103 ± 0.021 μg/mL). However, at the highest concentration (100 μg/mL) of PF (containing catechins and caffeine) a decrease in cell viability of both AML12 and HepG2 cells was observed as seen in Figure 3B. Cell viability of HepG2 and AML12 cells with treatment of 100 μg/mL NPF was more than 60% (Figure 3C). 0–25 μg/mL of GGL did not show any cytotoxic effect in HepG2 and AML12 cells (Figure 3D). In the resazurin reduction assays of combinations, cell viability decreased in AML12 cells with combinations of PF+NPF (each 12.5 μg/mL) or PF+GGL (each 12.5 μg/mL) (Figure 3). However, cytotoxicity was not observed, when PF+GGL (each 12.5 μg/mL) or PF+NPF+GGL (each 8.3 μg/mL) were treated in both cell lines (Figure 3).

### Microarray data

**Figure 4:**
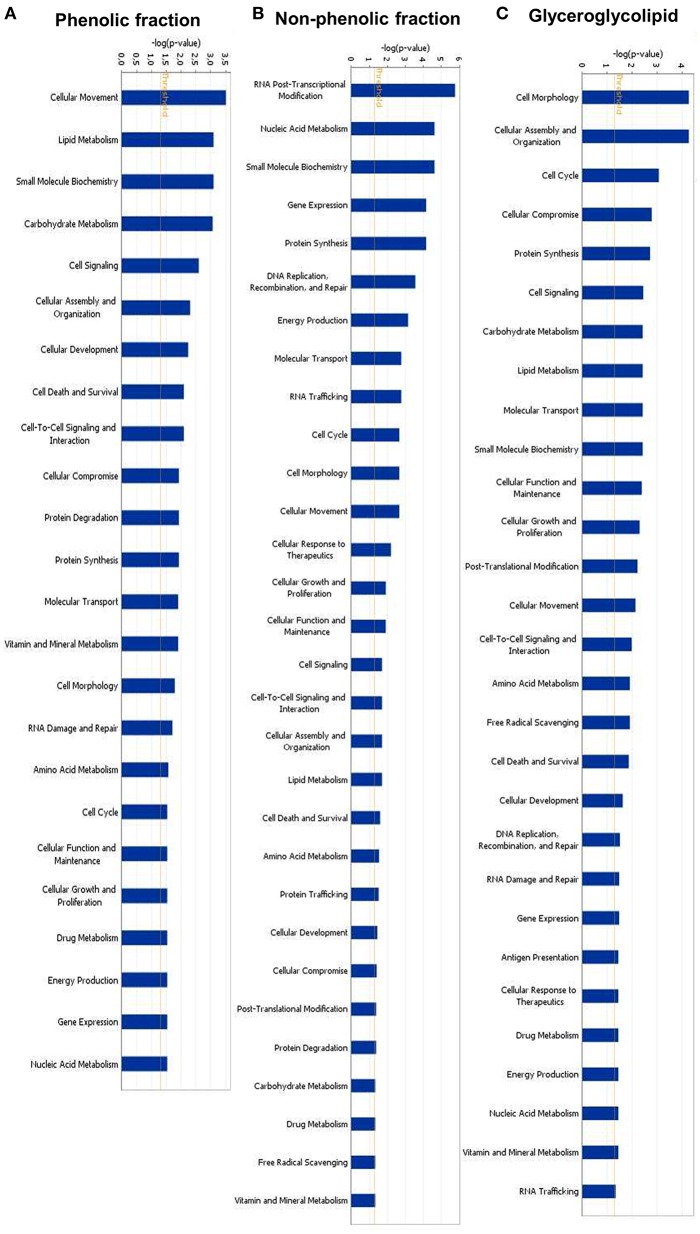
**Identified molecular functions of (A) phenolic fraction, (B) non-phenolic fraction, and (C) glyceroglycolipid of green tea by Ingenuity pathway analyses**.

We performed gene expression analysis to identify possible targets and mechanisms of action of green tea samples (PF, NPF, and GGL) in HepG2 cells. To get information about cellular functions, datasets were analyzed using the IPA Core Analysis tool. We identified three sets of genes differentially expressed upon treatment with the green tea samples. The genes were associated with cytoskeleton formation, cellular movement and morphology (Figure 4 and Tables 4, 5).

**Table 4:** **Top up- and down-regulated genes in HepG2 cells upon treatment of green tea samples (GGL, PF, or NPF) for 24 h**.

	**GGL**		**PF**		**NPF**	
	**Gene**	**FC^a^**	**Gene**	**FC^a^**	**Gene**	**FC^a^**
Top up-regulated genes	*UTF1*	11.3	*CD86*	13.2	*PCDH1*	158.7
	*CD86*	10.0	*DKKL1*	8.6	*MAL*	72.0
	*CCT3*	8.9	*DNAJC16*	8.5	*HDAC7*	65.8
	*CTTNBP2*	7.8	*ZNF365*	8.3	*DNAH2*	64.9
	*FHL1*	7.7	*NLRP1*	7.6	*MAPK8IP2*	57.7
	*ZNF365*	6.7	*CYP4X1*	7.3	*ARL3*	56.9
	*SYN1*	5.7	*SYN1*	7.1	*PELI2*	56.9
	*POLR2J2/POLR2J3*	5.5	*CLDN5*	6.6	*GBP4*	55.7
	*PRDX4*	5.5	*GPR179*	6.0	*NOX1*	53.4
	*MYH8*	5.3	*RCSD1*	5.5	*DENND2A*	52.0
Top down-regulated genes	*STK11*	−29.0	*VENTX*	−17.4	*TALDO1*	−1951.0
	*GPR126*	−18.3	*POT1*	−8.7	*LSM7*	−1562.9
	*GLDC*	−15.3	*PDZRN3*	−8.4	*NME2*	−1530.7
	*GSN*	−14.5	*PPAPDC1A*	−7.5	*HLA-G*	−1184.4
	*HNRNPM*	−13.9	*FHL1*	−7.0	*EEF1G*	−989.1
	*TMCO1*	−13.3	*SPO11*	−6.2	*NAB2*	−982.3
	*DNAJC7*	−13.3	*SMG7*	−5.4	*ATPIF1*	−897.6
	*RBM4*	−13.1	*NXN*	−5.2	*NDUFA8*	−861.1
	*GOLPH3*	−11.2	*TRPC2*	−5.0	*LOC645166*	−765.4
	SEC61G	−10.9	AKT3	−4.6	RPS15	−760.1

a *FC: fold change*.

*Fold changes of mRNA expression were detected by microarray hybridizations*.

**Table 5:** **Cell migration-associated genes deregulated in HepG2 cells after treatment with GGL**.

**Functions**	**Gene**	**Description**	**GGL**	**PF**	**NPF**
			**Fold change**		
Cellular movement	*HBEGF*	Heparin-binding EGF-like growth factor	−4.7	NC^a^	NC
	*TGFBR1*	Transforming growth factor, β-receptor 1	NC	2.5	NC
	*TGFBR2*	Transforming growth factor, β-receptor 2 (70/80 kDa)	−6.7	−2.8	NC
	*RAC1*	Ras-related C3 botulinum toxin substrate 1 (rho family, small GTP binding protein Rac1)	−3.2	NC	NC
	*MAP3K7*	Mitogen-activated protein kinase kinase kinase 7	−4.5	NC	NC
	*PIM1*	pim-1 oncogene	−4.9	NC	NC
	*RGS4*	Regulator of G-protein signaling 4	3.8	2.9	NC
	*MYC*	v-myc myelocytomatosis viral oncogene homolog (avian)	−8.3	−3.0	NC
	*PIM1*	pim-1 oncogene	−4.9	NC	NC
	*PFN1*	Profilin 1	−5.8	−2.8	NC
	*CDH11*	Cadherin 11, type 2, OB-cadherin (osteoblast)	NC	−2.5	NC
	*CSPG4*	Chondroitin sulfate proteoglycan 4	NC	−2.7	NC
	*PKD1*	Polycystic kidney disease 1 (autosomal dominant)	NC	−3.5	NC
	*AKT3*	v-akt murine thymoma viral oncogene homolog 3 (protein kinase B, γ)	NC	−4.6	NC
	*BMPR2*	Bone morphogenetic protein receptor, type II (serine/threonine kinase)	NC	2.5	NC
	*PRKCI*	Protein kinase C, 1	NC	−2.4	NC
	*PTP4A2*	Protein tyrosine phosphatase type IVA, member 2	NC	−2.8	NC
	*ADAM15*	ADAM metallopeptidase domain 15	NC	3.2	NC
	*MYH10*	Myosin, heavy chain 10, non-muscle	NC	3.1	NC
	*STK11*	Serine/threonine kinase 11	−29.0	NC	−308.7
	*NME1*	NME/NM23 nucleoside diphosphate kinase 1	NC	NC	−333.1
	*YBX1*	Y box binding protein 1	NC	NC	−84.4
	*ARL3*	ADP-ribosylation factor-like 3	NC	NC	56.886
	*AURKA*	Aurora kinase A	NC	NC	−70.5
	*BECN1*	Beclin 1, autophagy related	NC	NC	−84.4
	*CIT*	Citron (rho-interacting, serine/threonine kinase 21)	NC	NC	−72.0
	*NUSAP1*	Nucleolar and spindle associated protein 1	NC	NC	−58.5
	*RHOA*	Ras homolog family member A	NC	NC	−82.1
	*TM4SF1*	Transmembrane 4 L six family member 1	NC	NC	−69.1
	*TTC19*	Tetratricopeptide repeat domain 19	NC	NC	−78.8
	*FOXP1*	Forkhead box P1	NC	NC	−124.5
	*DEK*	DEK oncogene	NC	NC	−40.8
	*GDF15*	Growth differentiation factor 15	NC	NC	−57.7
	*CCL20*	Chemokine (C-C motif) ligand 20	NC	NC	−116.2
	*C1QBP*	Complement component 1, q subcomponent binding protein	NC	NC	−45.3
	*PGF*	Placental growth factor	NC	NC	−259.6
	*BAG*	BCL2-associated athanogene	NC	NC	−90.5
Cytoskeleton formation	*ARF6*	ADP-ribosylation factor 6	−3.4	NC	NC
	*ARHGEF10*	Rho guanine nucleotide exchange factor (GEF) 10	3.4	NC	NC
	*ARHGEF3*	Rho guanine nucleotide exchange factor (GEF) 3	−2.9	NC	NC
	*GNB1*	Guanine nucleotide binding protein (G protein), β-polypeptide 1	−4.0	NC	NC
	*GSN*	Gelsolin	−14.5	NC	NC
	*NCK1*	NCK adaptor protein 1	−2.9	NC	NC
	*PTPN11*	Protein tyrosine phosphatase, non-receptor type 11	−3.7	NC	NC
	*TGFBR2*	Transforming growth factor, beta receptor 2 (70/80kDa)	−6.7	NC	NC
	*MAP3K7*	Mitogen-activated protein kinase kinase kinase 7	−4.5	NC	NC
Morphology	*GOLPH3*	Golgi phosphoprotein 3 (coat-protein)	−11.2	−2.8	NC
	*HMGA1*	High mobility group AT-hook 1	−2.9	NC	NC
	*LETM1*	Leucine zipper-EF-hand containing transmembrane protein 1	2.9	2.5	NC
	*PRDX3*	Peroxiredoxin 3	−3.1	NC	NC
	*SEC23IP*	SEC23 interacting protein	−5.4	NC	NC
	*MT1F*	Metallothionein 1F	−6.2	−3.1	NC
	*KLF2*	Kruppel-like factor 2 (lung)	−3.8	NC	NC
	*PIKFYVE*	Phosphoinositide kinase, FYVE finger containing	−3.8	−2.6	NC
	*LZTS2*	Leucine zipper, putative tumor suppressor 2	−4.8	NC	NC
	*BST2*	Bone marrow stromal cell antigen 2	−5.8	NC	−286.0
	*BHLHE40*	Basic helix-loop-helix family, member e40	−3.1	NC	NC
	*TMEM123*	Transmembrane protein 123	−9.1	NC	NC
	*ABL1*	c-abl oncogene 1, non-receptor tyrosine kinase	−2.9	NC	NC
	*EP300*	E1A binding protein p300	−3.2	NC	NC
	*CTSZ*	Cathepsin Z	NC	−3.9	NC
	*PRDX1*	Peroxiredoxin 1	NC	−2.8	NC
	*GPR18*	G protein-coupled receptor 18	NC	5.2	NC
	*GPR182*	G protein-coupled receptor 182	NC	5.1	NC
	*MMP10*	Matrix metallopeptidase 10 (stromelysin 2)	NC	−2.4	NC
	*ARHGEF10*	Rho guanine nucleotide exchange factor (GEF) 10	3.4	NC	NC
	*ARHGEF3*	Rho guanine nucleotide exchange factor (GEF) 3	−2.9	NC	NC
	*GNB1*	Guanine nucleotide binding protein (G protein), β-polypeptide 1	−4.0	NC	NC
	*GSN*	Gelsolin	−14.5	NC	NC
	*NCK1*	NCK adaptor protein 1	−2.9	NC	NC
	*PTPN11*	Protein tyrosine phosphatase, non-receptor type 11	−3.7	NC	NC
	*SMARCA4*	SWI/SNF related, matrix associated, actin dependent regulator of chromatin, subfamily a,	NC	NC	−132.5
	*TGFBR2*	Transforming growth factor, β-receptor 2 (70/80kDa)	−6.7	NC	NC
	*MAP3K7*	Mitogen-activated protein kinase kinase kinase 7	−4.5	NC	NC
	*PIM1*	Pim-1 oncogene	−4.9	NC	NC
	*KPNA2*	Karyopherin α 2 (RAG cohort 1, importin α 1)	NC	NC	−39.9
	*NTM1*	Nucleophosmin (nucleolar phosphoprotein B23, numatrin)	NC	NC	−56.1
	*CTTN*	Cortactin	NC	NC	−126.2
	*CSE1L*	CSE1 chromosome segregation 1-like (yeast)	NC	NC	−109.1
	*RTN4*	Reticulon 4	NC	NC	−132.5
	*LGALS3*	Lectin, galactoside-binding, soluble, 3	NC	NC	−186.1
	*TYMS*	Thymidylate synthetase	NC	NC	−181.0

a *NC: no change*.

### Validation of RNA expression by real-time RT-PCR

The microarray results were exemplarily validated by real-time RT-PCR experiments and comparable results were obtained (Table 6). The correlation coefficients between mRNA expression values determined by microarray and real time RT-PCR were *R* = 0.94 (Pearson correlation test). This indicated a high degree of concordance between the results obtained by both methods.

**Table 6:** **Validation of microarray-based mRNA expression by quantitative real-time RT-PCR (RT-PCR were performed twice)**.

**Cell line**	**Gene**	**Method**	**Samples**	**FC**	**Samples**	**FC**
HepG2	*CD86*	Microarray hybridization	GGL	9.99	PF	13.18
		Real-time RT-PCR		5.19		9.57
HepG2	*ZNF365*	Microarray hybridization	GGL	6.68	PF	8.28
		Real-time RT-PCR		1.49		1.53
HepG2	*SYN1*	Microarray hybridization	GGL	5.69	PF	7.06
		Real-time RT-PCR		2.18		2.56
HepG2	*STK11*	Microarray hybridization	GGL	−29.04	NPF	−308.69
		Real-time RT-PCR		−1.00		−16.32

*R-value: 0.94 (Pearson correlation test)*.

### Inhibition of cell migration and effect on microtubule dynamics

**Figure 5:**
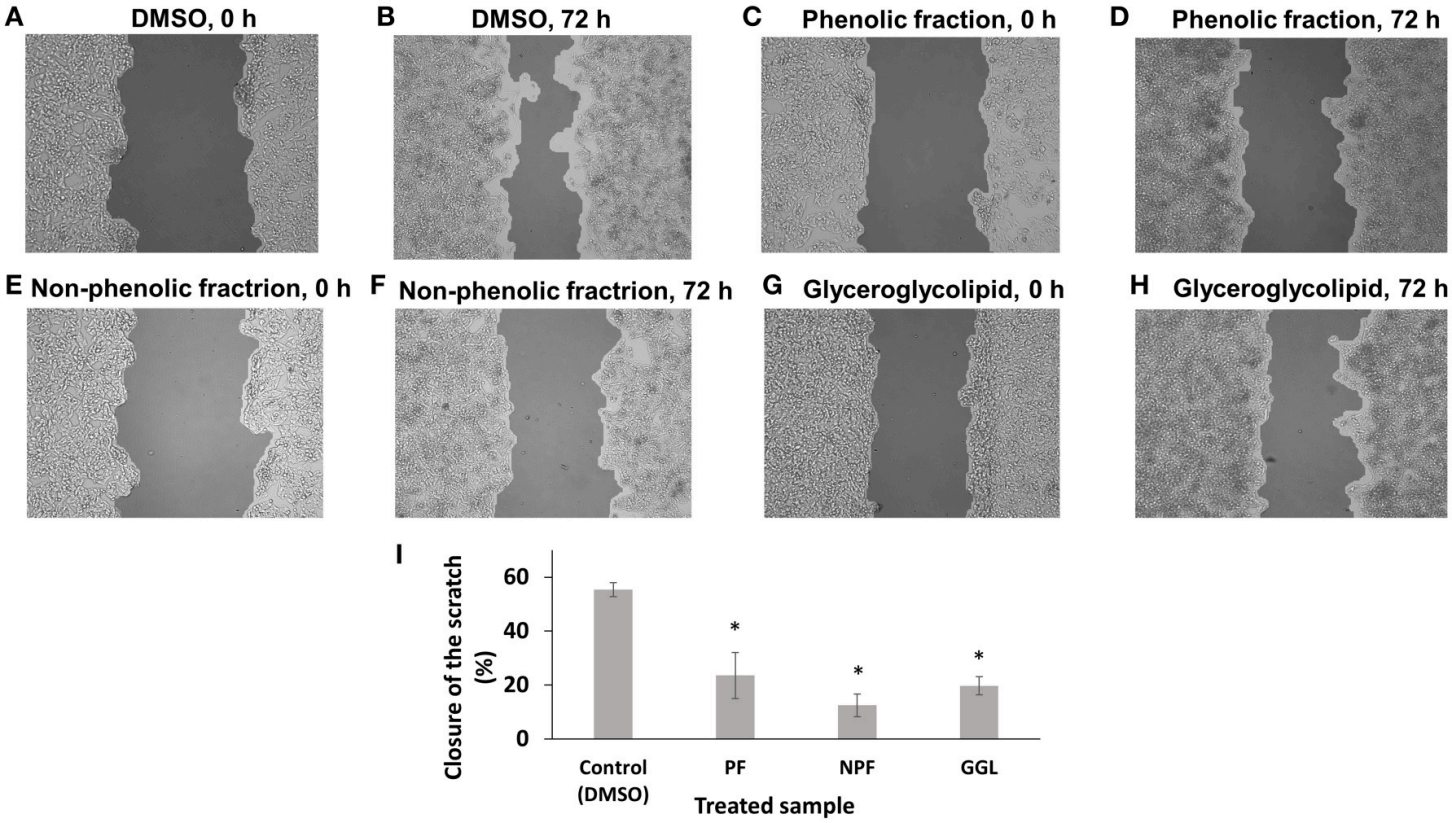
**Inhibition of migration of HepG2 cells by green tea samples**. HepG2 cells were treated with **(A)** DMSO for 0 h, **(B)** DMSO for 72 h, **(C)** phenolic fraction for 0 h, **(D)** phenolic fraction for 72 h, **(E)** non-phenolic fraction for 0 h, **(F)** non-phenolic fraction for 72 h, **(G)** glyceroglycolipid for 0 h, **(H)** glyceroglycolipid for 72 h, **(I)** Quantification of closure of the scratch by TScratch software. Significantly different according to Student's *t*-test, ^*^P ≤ 0.05.

**Figure 6:**
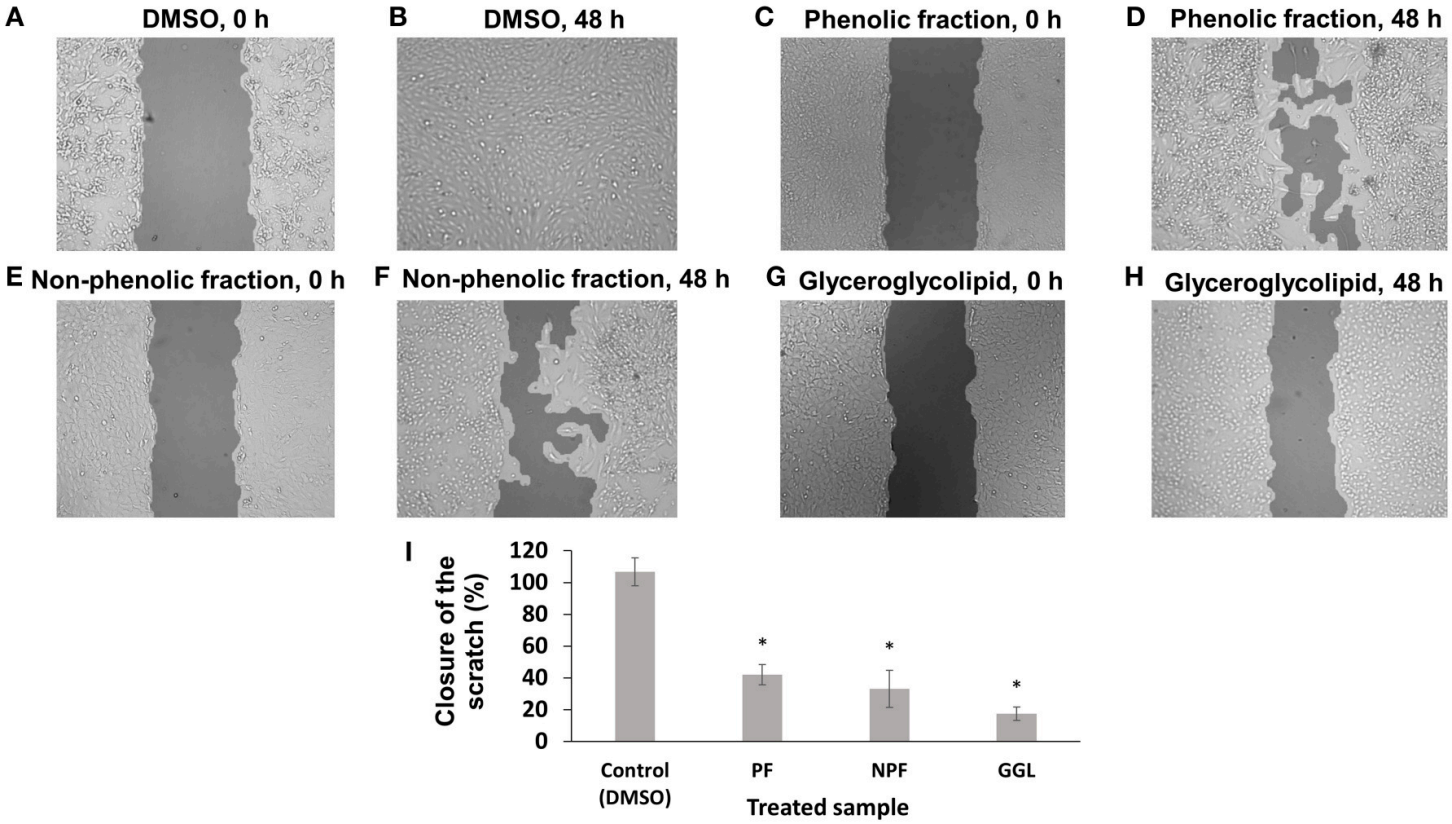
**Inhibition of migration of U2OS-GFP-α-tubulin cells by green tea samples**. U2OS-GFP-α-tubulin cells were treated with **(A)** DMSO for 0 h, **(B)** DMSO for 72 h, **(C)** phenolic fraction for 0 h, **(D)** phenolic fraction for 72 h, **(E)** non-phenolic fraction for 0 h, **(F)** non-phenolic fraction for 72 h, **(G)** glyceroglycolipid for 0 h, **(H)** glyceroglycolipid for 72 h, **(I)** Quantification of closure of the scratch by TScratch software. Significantly different according to Student's *t*-test, ^*^P ≤ 0.05.

Since we identified genes associated with cytoskeleton formation, cellular movement, and morphology, we assumed that these green tea samples may inhibit cell motility. Therefore, we performed scratch migration assays with HepG2 and U2OS cells (Figures 5, 6). Each cell has different characteristics. HepG2 cells grow slowly and form a clump of cells instead of spreading cells while cells are growing. Therefore, HepG2 cell layers were not completely closed with the treatment of DMSO, the negative control, after incubation for 72 h. HepG2 cell layers treated with green tea extracts for 72 h showed interrupted closures in contrast to treatment with DMSO (Figure 5). After treatment of GGL, PF, and NPF for 72 h, HepG2 cell layers were closed only by 19, 23, and 12% of the initial scratched areas, respectively, whereas HepG2 cell layer treated with DMSO for 72 h were closed by 55% of the initial scratch width (Figure 5).

**Figure 7:**
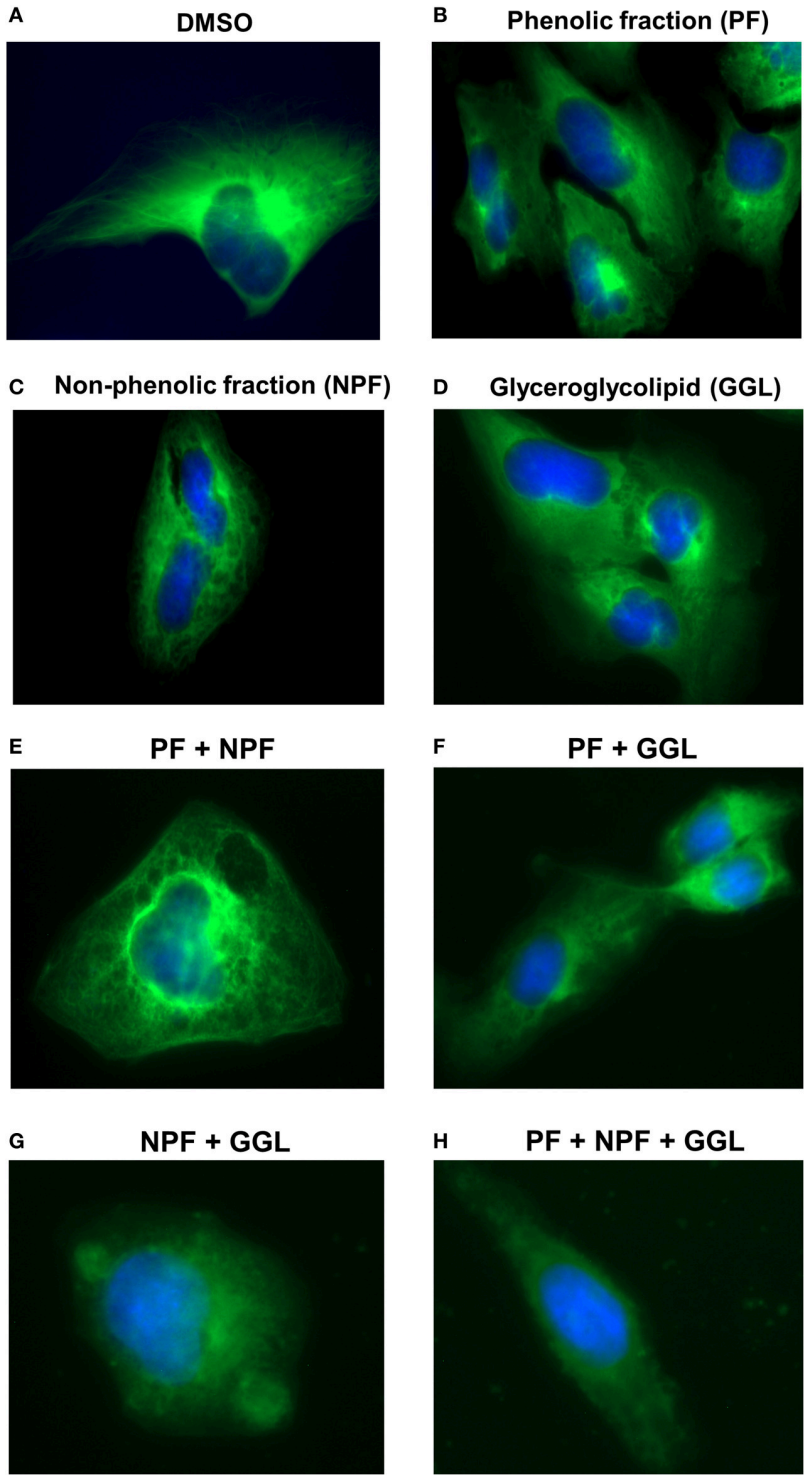
**Live cell imaging of U2OS-GFP-α-tubulin cells stably transfected with a GFP fusion construct of α-tubulin cells and treated with (A) DMSO or 25 μg/mL of green tea samples [(B) PF, (C) NPF, or (D) GGL], combinations of (E) PF+NPF (each 12.5 μg/mL), (F) PF+GGL (each 12.5 μg/mL), (G) NPF+GGL (each 12.5 μg/mL), and (H) PF+NPF+GGL (each 8.3 μg/mL)**.

To investigate the effects of the green tea samples in more details, we used U2OS cells stably transfected with a GFP fusion construct of α-tubulin for scratch assays as well as observation of microtubule dynamics (Figures 6, 7). In contrast to HepG2 cells, U2OS cells grow fast and are spreading, when they grow. U2OS cell layer treated DMSO as solvent control showed a complete scratch closure in most case after 48 h. However, cell layer treated with each 25 μg/mL of the three green tea samples showed significantly delayed closures of the scratches. Only 17% of the initial scratch width was closed up on treatment with GGL after 48 h, while 42% and 33% of the initial scratch width were recolonized upon treatment with PF and NPF after 48 h, respectively (Figure 6). Our scratch assays with two different cell lines clearly demonstrated that green tea samples inhibit cell migration of both cell lines.

Microtubules are indispensable for the directional migration of cells (Watanabe et al., 2005). Since our gene expression profiling showed a high number of deregulated genes associated with microtubule cytoskeleton, we treated U2OS-GFP-α-tubulin cells with 25 μg/mL of the samples or combinations of three samples [PF+NPF (each 12.5 μg/mL), PF+GGL (each 12.5 μg/mL), NPF+GGL (each 12.5 μg/mL), PF+NPF+GGL (each 8.3 μg/mL)], and analyzed their effect on microtubule activity. The number of distinct tubulin filaments decreased upon treatment with 25 μg/mL of each green tea sample for 2 h (Figure 7). We also observed significantly decreased tubulin filaments after treatment of all combined samples for 2 h (Figure 7).

## Discussion

The aim of this study was to investigate the effects of green tea constituents in human cancer cells. Similar to previous studies, we also found abundant catechins in the phenolic fraction (PF), e.g., 46.9% EGCG. NPF was also analyzed by different chromatographic procedures and six glyceroglycolipids were identified with an abundance of up to 2% in this fraction. One of the pure glyceroglycolipid, GGL (0.142% of NPF) was characterized as 3-[(1-oxohexadecyl)oxy]-2-[(1-oxooctadecyl)oxy]propyl-6-*O*-α-D-galactopyranosyl β-D-galactopyranoside by NMR and mass data. This compound was selected for further mechanistic study. The glyceroglycolipids in green tea were identified and reported earlier (Ali et al., 2010), but to the best of our knowledge there is no report of their chemopreventive action in green tea. While EGCG and other catechins in green tea attracted much attention in the past years, non-phenolic compounds in this plant have been largely neglected. Therefore, we investigated the effects of nonphenolic components of green tea (NPF) and one of the isolated GGLs toward human cancer and compared with the effects of well-known PF containing catechins.

It has been reported that glyceroglycolipids showed tumor suppressive effects (Murakami et al., 1995; Hou et al., 2007). Hou et al. identified the bioactive glyceroglycolipid 1,2-di-*O*-α-linolenoyl-3-*O*-β-galactopyranosyl-sn-glycerol (DLGG) from *Crassocephalum rabens* (Asteraceae), which is a popular herbal medicine and food supplement in Taiwan for various inflammation-related syndromes and this glyceroglycolipid can suppress NF-κB and its downstream inflammatory mediators, NO, iNOS, COX-2, and prostaglandin E_2_, *in vitro* (Hou et al., 2007). The anti-inflammatory effects of this glyceroglycolipid might be also responsible for its significant cancer chemopreventive activity (Hou et al., 2007). Besides, two glyceroglycolipids were isolated from the leaves of *Citrus hystrix* (bitter orange), a traditional herb in Thailand (Murakami et al., 1995). They were identified as 1,2-di-*O*-α-linolenoyl-3-*O*-β-galactopyranosyl-*sn*-glycerol (DLGG, 1) and a mixture of two compounds, 1-*O*- α-linolenoyl-2-*O*-palmitoyl-3-*O*-β-galactopyranosyl-*sn*-glycerol (2a) and its counterpart (2b) (LPGG, 2). Both lipids were potent inhibitors of tumor promoter-induced Epstein-Barr virus (EBV) activation (Murakami et al., 1995). Therefore, we hypothesized that glyceroglycolipids in green tea, which have not been investigated yet, may have anti-tumor effects, therefore, they are worth to be tested.

Several biological properties have been reported for green tea, including the prevention of cancer and cardiovascular diseases, as well as anti-inflammatory, anti-arthritic, antibacterial, antiangiogenic, anti-oxidative, antiviral, neuroprotective, and cholesterol-lowering effects (Haqqi et al., 1999; Kavanagh et al., 2001; Osada et al., 2001; Sueoka et al., 2001; Sartippour et al., 2002; Donà et al., 2003; Raederstorff et al., 2003; Weber et al., 2003; Sudano Roccaro et al., 2004; Weinreb et al., 2004). Polyphenolic catechins were identified as active ingredients (Bettuzzi et al., 2006; Chacko et al., 2010). In a double-blind placebo-controlled study, green tea catechins were safe and effective for treating premalignant prostate cancer (Bettuzzi et al., 2006). Besides, clinical activity of green tea was shown against prostate cancer (Jatoi et al., 2003; Choan et al., 2005). Case-control studies supported the protective effect of green tea against prostate, esophageal, colon, rectum and pancreatic cancers (Ji et al., 1997; Jian et al., 2004).

In lieu of a number of previous studies reporting the chemopreventive effects of green tea (Ji et al., 1997; Arteel et al., 2002; Jian et al., 2004; Abe et al., 2005, 2007), we investigated possible targets and mechanisms of action using microarray analysis of green tea sample treated HepG2 hepatocellular carcinoma cells. We found that the green tea samples at a non-toxic or weakly cytotoxic concentration (25 μg/mL) affected molecular functions of cell morphology and cellular movement and several cell migration-associated genes were identified. PF, rich in catechins, deregulated several genes associated with cellular movement and cell morphology functions. NPF deregulated a number of genes related to cellular movement and cell morphology with high fold changes. GGL isolated from NPF was also effective in changing cell morphology and cellular movement. Interestingly, it also deregulated genes related to cytoskeleton formation.

Since deregulation of cell migration-associated genes was shown for all three green tea samples, we validated our microarray data by the scratch migration assay and fluorescence microscopy of microtubule dynamics. Indeed, cell migration was significantly inhibited and the number of tubulin filaments were decreased by all three samples. These results indicate that green tea inhibit cell migration by the disruption of microtubule cytoskeleton. These data indicate that not only PF of green tea acts in a chemopreventive manner, but also the NPF with GGL as one of its ingredients. As of yet, the filamentous proteins did not attract much attention as possible targets for chemoprevention by green tea.

Cytoskeletal elements such as tubulins, keratins, vimentin, desmin, actin, and others represent widely distributed proteins in eukaryotic cells with crucial functions for cell morphology, motility, division, etc. (Lowery et al., 2015). Interestingly, cytoskeletal proteins are also involved in signal transduction and oncogenic signaling (Prendergast and Gibbs, 1993; Rao and Li, 2004; Jiang et al., 2009). Lu et al. (2005) reported that green tea extract modulated actin remodeling in a multi-step carcinogenesis model. These results fit well to our observation that PF, NPC, and GGL all affect cellular morphology and movement as well as microtubule formation. Based on the results obtained by us and Lu et al. (2005), it is reasonable to hypothesize that cytoskeletal proteins may represent interesting targets for chemoprevention and cancer therapy by green tea.

In our study, we observed that green tea samples were not very toxic toward both HepG2 cancer cells and AML12 normal hepatocytes. Cell viability was decreased with combinations of PF+NPF, PF+GGL in AML12 cells, however, combination of all three samples did not show any cytotoxic effect in both cells. It is remarkable that the inhibition of cellular movement, migration, and microtubule formation took place at concentrations, which were not or only minimally cytotoxic against both liver cancer cells and normal hepatocytes. Therefore, it can be expected that these green tea extracts would not exert considerable toxic side effects in normal tissues of cancer patients. Having in mind the tremendous life-threatening side effects of most chemotherapeutic drugs, it would be desirable to have anti-invasive and anti-metastatic drugs available that are safe and tolerable without severe side effects.

In conclusion, we identified not only PF, but also a glyceroglycolipid in NPF as contributing factor to the chemopreventive effects of green tea. Both PF and NPF of green tea inhibited cancer cell migration by the disassembly of microtubules, even though they were not cytotoxic. Hence, green tea may have a high potential for application in the prevention of human cancers.

## Author contributions

ES performed resazurin assay, real-time reverse transcription PCR and scratch migration assay. CW evaluated microarray data. ZA and YW performed phytochemical analyses. SK, LW, and IK supervised phytochemical analyses. TE designed the paper and ES and TE wrote the paper.

### Conflict of interest statement

The authors declare that the research was conducted in the absence of any commercial or financial relationships that could be construed as a potential conflict of interest.

## Abbreviations

8-OhdG: 8-hydroxydeoxy-2′-deoxyguanosine
EGCG: epigallocatechin-3-gallate
GGL: glyceroglycolipid
NPF: non-phenolic fraction
PF: phenolic fraction
GFP: green fluorescence protein.
